# Optical and microstructural properties of ZnO/TiO_2_ nanolaminates prepared by atomic layer deposition

**DOI:** 10.1186/1556-276X-8-107

**Published:** 2013-02-27

**Authors:** Yu-Zhu Gu, Hong-Liang Lu, Yang Geng, Zhi-Yuan Ye, Yuan Zhang, Qing-Qing Sun, Shi-Jin Ding, David Wei Zhang

**Affiliations:** 1 State Key Laboratory of ASIC and System, Department of Microelectronics, Fudan University, Shanghai 200433, China

**Keywords:** ZnO/TiO_2_ nanolaminates, ALD, Transmittance, HRTEM

## Abstract

ZnO/TiO_2_ nanolaminates were grown on Si (100) and quartz substrates by atomic layer deposition at 200°C using diethylzinc, titanium isopropoxide, and deionized water as precursors. All prepared multilayers are nominally 50 nm thick with a varying number of alternating TiO_2_ and ZnO layers. Sample thickness and ellipsometric spectra were measured using a spectroscopic ellipsometer, and the parameters determined by computer simulation matched with the experimental results well. The effect of nanolaminate structure on the optical transmittance is investigated using an ultraviolet–visible-near-infrared spectrometer. The data from X-ray diffraction spectra suggest that layer growth appears to be substrate sensitive and film thickness also has an influence on the crystallization of films. High-resolution transmission electron microscopy images show clear lattice spacing of ZnO in nanolaminates, indicating that ZnO layers are polycrystalline with preferred (002) orientation while TiO_2_ layers are amorphous.

## Background

ZnO is a low-cost and widely used semiconductor material with outstanding physical and chemical characteristics. At room temperature, the band gap and exciton binding energy of ZnO are 3.37 eV and 60 meV, respectively, both contributing to its extraordinary chemical and thermal stability. Thus, ZnO thin films exhibit magnificent applications in the manufacturing process of optoelectronic devices [1]. Also, being a promising semiconductor material that is transparent to visible light and has excellent optical transmittance, TiO_2_ is widely used in the synthesis of semiconductor photocatalysts, solar cell electrodes, and sophisticated electronic optical devices [2-5].

ZnO and TiO_2_ thin films, both with a wide band gap, high refractive index, high stability, and good catalysis, are suitable partners for multilayer nanostructures. On the one hand, TiO_2_ could serve as a buffer layer between ZnO and Si substrates. The lattice and thermal mismatches can be reduced, and the quality of ZnO films will be enhanced because TiO_2_ can inhibit the surface silicon atoms from plundering oxygen atoms in ZnO films [6,7]. Moreover, growing very thin ZnO films over a porous TiO_2_ electrode can improve the surface state and surface atomic mobility, so high-powered solar cells with better utilization efficiency can be produced [8]. There are also researches on ZnO/TiO_2_ multilayer mirrors at ‘water-window’ wavelengths with high reflectivity around 2.7 nm, indicating its potential in multilayer optics [9].

ZnO/TiO_2_ multilayers have been prepared by many techniques, such as chemical vapor deposition, pulsed laser deposition, and co-sputtering [10-12]. However, high-quality nanolaminate films require precisely controlled factors including interfacial roughness, interdiffusion between layers, layer-to-layer consistency, and conformality. Atomic layer deposition (ALD) is more powerful in preparing such multilayers than other techniques, which keeps the precursors separated during the reaction [13]. By sequentially dosing the surface with appropriate chemical precursors and then promoting surface reactions that are inherently self-limiting, the atomic layer control of film growth can be obtained. There has been a variety of publications on ALD-prepared ZnO or TiO_2_ films [14-17]. Thus, studies on ZnO/TiO_2_ multilayers prepared by ALD are of increasing importance in this field [18,19]. In this study, a series of ZnO/TiO_2_ nanolaminates were prepared by ALD. The optical and microstructural properties of ZnO/TiO_2_ were measured and compared by spectroscopic ellipsometry (SE), ultraviolet–visible-near-infrared (UV–vis-NIR) spectrometry, X-ray diffraction (XRD), and high-resolution transmission electron microscopy (HRTEM).

## Methods

**Figure 1:**
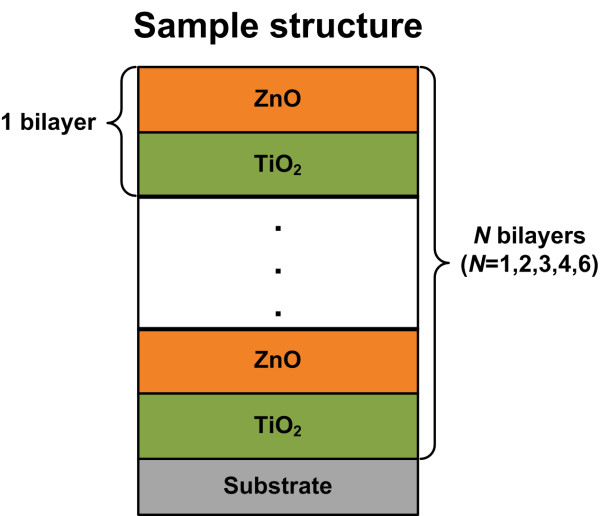
**Schematic of physical models of ZnO/TiO**_**2 **_**nanolaminates grown by ALD.**

ZnO/TiO_2_ multilayers were deposited at 200°C using a BENEQ TFS-200 ALD reactor (Beneq Oy, Vantaa, Finland) on n-doped Si (100) (*ρ* = 1 to 10 Ω cm) and quartz substrates. ZnO films were deposited by alternating exposures to diethylzinc (DEZn) and deionized (DI) water, while TiO_2_ films were prepared using titanium isopropoxide (TTIP) and DI water as precursors. The TTIP and DEZn were held in stainless bubblers at 58°C and 18°C, respectively. The precursors were alternately introduced to the reactor chamber using high-purity N_2_ (>99.99%) as a carrier gas. An ALD cycle of TiO_2_ films consisted of 1.0-s TTIP dosing, 5.0-s N_2_ purge, 0.5-s DI water dosing, and 5.0-s N_2_ purge, while for ZnO films, the cycle is 0.5-s DEZn/2.0-s N_2_/0.5-s DI water/2.0-s N_2_. A schematic of five sample structures is given in Figure 1. Multilayers were prepared in depositing alternating layers of TiO_2_ and ZnO. Five samples contain one, two, three, four, and six ZnO/TiO_2_ bilayers, respectively. Each structure was deposited on Si and quartz substrates, respectively, so ten samples were prepared actually. The nominal film thickness for the multilayer was 50 nm.

The thicknesses of the multilayer were measured by spectroscopic ellipsometry (Sopra GES5E, SOPRA, Courbevoie, France) where the incident angle was fixed at 75° and the wavelength region from 230 to 900 nm was scanned with 5-nm steps. The optical transmission spectra were obtained using a UV spectrophotometer (UV-3100) in a wavelength range of 200 to 900 nm at room temperature in air. The crystal structures of the films were obtained using an X-ray diffractometer (D8 ADVANCE, Bruker AXS, Inc., Madison, WI, USA) using Cu Kα radiation (40 kV, 40 mA, *λ* = 1.54056 Å). High-resolution transmission electron microscopy and electron diffraction experiments were performed in a Philips CM200-FEG system operated at 200 kV. The specimens were prepared by mechanical polishing and dimpling, followed by Ar^+^ ion milling to electron transparency with 4.0-keV beam energy at an angle of 6° using a Gatan precision ion polishing system (Pleasanton, CA, USA).

## Results and discussion

**Figure 2:**
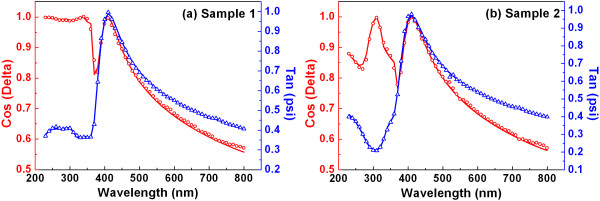
**Comparison of experimental (open symbol) and calculated (solid line) ellipsometric spectra (cosΔ and tanψ).** (**a**) Sample 1. (**b**) Sample 2.

The experimental and fitted ellipsometric spectra of ZnO/TiO_2_ multilayer thin films were measured using the spectroscopic ellipsometer. For example, the experimental (open symbol) and calculated (solid line) ellipsometric spectra (cosΔ and tanψ) of samples 1 and 2 are presented in Figure 2a,b, respectively. It can be observed that the experimental and fitting curves match very well, with the accuracy of the regression (*R*^2^) greater than 0.998. Table 1 shows the layer thickness of the samples grown on Si substrate. As can be seen, total thicknesses for samples 1 to 5 are 51.14, 48.27, 46.92, 46.56, and 46.47 nm, respectively. The thickness of the first sample with single bilayer is very close to the nominal thickness of 50 nm. However, with the increase of TiO_2_ layers, the total thickness seems to be slightly thinner than the expected one, resulting from the reduced adsorption of DEZn on TiO_2_.

**Table 1:** The measured layer thickness of films with indexes 1 to 5 grown on Si by SE

**Sample ID**	**1**	**2**	**3**	**4**	**5**
1st layer-TiO_2_	18.85	8.85	5.87	4.23	2.73
1st layer-ZnO	32.29	15.13	10.67	7.49	5.31
2nd layer-TiO_2_		8.97	4.81	4.15	2.47
2nd layer-ZnO		15.32	10.37	7.46	5.28
3rd layer-TiO_2_			4.87	4.13	2.39
3rd layer-ZnO			10.33	7.41	5.32
4th layer-TiO_2_				4.24	2.38
4th layer-ZnO				7.45	5.28
5th layer-TiO_2_					2.38
5th layer-ZnO					5.29
6th layer-TiO_2_					2.36
6th layer-ZnO					5.28
Total thickness (nm)	51.14	48.27	46.92	46.56	46.47

**Figure 3:**
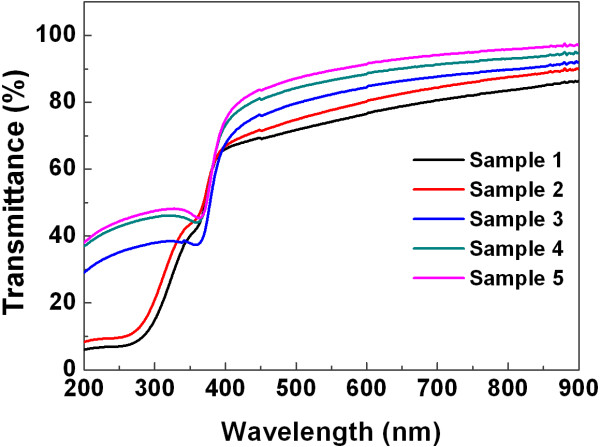
**Transmittance spectrum of ZnO/TiO**_**2 **_**nanolaminates.**

Transmittance spectrum for the samples grown on quartz is given in Figure 3. It can be found that the average transmittance over the entire visible wavelength range of 400 to 900 nm is more than 75%, while a strong absorption peak appears at 380 nm near the ultraviolet region. The transmittance increases with the decrease of the thickness of each TiO_2_ and ZnO layer. Moreover, the spectral transmittance value intensively decreases with the photon energy in the ultraviolet region. This is due to the strong absorption from fundamental band gap and high-energy critical point transitions. Since the emission band of ZnO is near the UV region, we can assume that the peak is a free-exciton absorption peak caused by oxygen vacancies in the film. It should be noted that the transmittances of samples 1 and 2 incline to 8% in the UV region, while the last three samples exhibit much higher transmittance, all between 30% and 40%. It suggests that the absorption in the UV region significantly depends on the sample structure. As the sample ID number increases, each ZnO layer in the sample becomes thinner, comparted by more TiO_2_ films, which prevents photon from being fully absorbed by ZnO, that is why the spectra drift upwards in the UV region [20-22].

**Figure 4:**
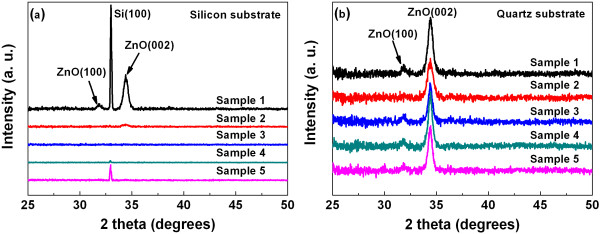
**XRD spectra of ZnO/TiO**_**2 **_**nanolaminates.** (**a**) Si substrate. (**b**) Quartz substrate.

Figure 4a,b shows the XRD patterns of as-deposited ZnO/TiO_2_ nanolaminates on Si and quartz substrates, respectively. For sample 1 grown on Si substrate, XRD peaks appear at 2*θ* = 31.8° and 34.4°, which correspond with the spacing in (100) and (002) directions of the ZnO layer, respectively. However, only a small (002) peak is observed in sample 2, while no obvious peaks are observed in the other samples, which suggests that ZnO crystallization is suppressed with ZnO films getting thinner. So ZnO peaks could only be observed in the first two samples, where the thickness of a single ZnO layer is over 15 nm. On the other hand, a strong (002) peak along with (100) is observed for all the samples deposited on quartz. Strong (002) preferential orientation indicates the polycrystalline nature of the ZnO layer. ZnO grains are mainly (002)-aligned corresponding to the wurtzite structure of ZnO [23]. It suggests that ZnO layers within multilayers were grown on amorphous TiO_2_ layers and showed preferred (002) orientation. In addition, no TiO_2_ phase is detected in all samples. Taken together, these data suggest that layer growth appears to be substrate sensitive and film thickness also has an influence on the crystallization of films.

**Figure 5:**
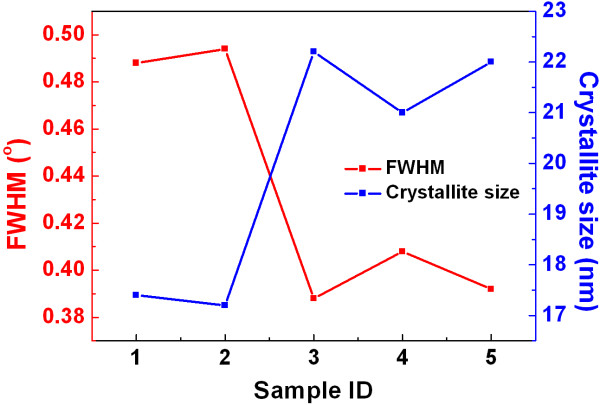
FWHM of (002) peaks and average grain sizes for ZnO films deposited on quartz substrates.

For further investigation, the lattice constants of ZnO films grown on quartz are calculated according to Bragg’s law [24]:

(1) d=λ/2sinθ,

where *d* is the interplanar spacing, *λ* is the X-ray wavelength which equals to 1.54 Å for Cu Kα radiation in this case, and *θ* is the scattering angle. Thus, the calculated values of *d* for ZnO (100) and (002) orientations are 2.8 and 2.6 Å, respectively. The grain size (*D*) of each ZnO layer can also be estimated using the Scherrer formula:

(2) D=Kλ/βcosθ,

where *D* is the average crystallite size, *K* (=0.89) is a constant, *λ* is the wavelength (Å), *β* is the full width at half maximum (FWHM) of peaks, and *θ* is the Bragg angle [25]. Figure 5 shows the FWHM values and average grain sizes for ZnO (002) films on quartz substrates. It can be seen that the grain sizes for the first two samples are around 17 nm, while this value rises to 21 nm for the next three samples. The tendency coincides with the observed increase of transmittance above.

**Figure 6:**
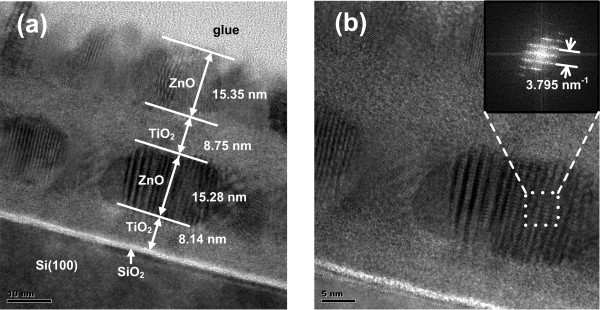
**High-resolution TEM images (a, b) of the four-layer ZnO/TiO**_**2 **_**nanolaminate on Si (100) substrate.** Inset shows FFT image of ZnO layer.

The cross-sectional HRTEM image of the ZnO/TiO_2_ nanolaminate is presented in Figure 6. We took the second sample on Si substrate representatively for analysis. As shown in Figure 6a, the ZnO/TiO_2_ nanolaminate film is well prepared by ALD. The comparatively dark layers are ZnO layers, and the other two gray layers are TiO_2_ layers. In addition, a bright layer is also found between the first TiO_2_ layer and the substrate, which is a SiO_2_ interfacial layer, because the Si substrate is slightly oxidized during the ALD process. Furthermore, the thicknesses for TiO_2_ and ZnO layers are respectively detected, which are consistent with the results measured from SE. However, the thickness of the first TiO_2_ layer is slightly thinner than expected. It is mainly because growth rate was unsteady at the beginning of the ALD process. In addition, as referred above, the formed interfacial SiO_2_ layer between TiO_2_ and Si substrate will snatch oxygen atoms and decrease the growth rate of TiO_2_.

Crystallized ZnO shows clear lattice in the image, while a crystal structure could hardly be observed in TiO_2_ layers. Thus, TiO_2_ films are amorphous, that is why no diffraction peaks are observed in XRD. Fast Fourier transformation (FFT) image is shown in the HRTEM image (Figure 6b). The reciprocal lattice spacing can be identified to be 3.795 nm^−1^. As a result, the interplanar spacing is 2.6 Å, which is consistent with the calculated data for ZnO (002) orientation. Thus, it could be concluded that ZnO films grow on TiO_2_ along the (002) direction [26,27]. Besides, the crystallite size of ZnO film shown in TEM images is also very close to the values calculated from XRD peaks, further confirming the structure features of ZnO/TiO_2_ nanolaminate.

## Conclusions

ZnO/TiO_2_ nanolaminates were grown on Si (100) and quartz substrates by ALD technique at 200°C. The optical and microstructural properties of samples with different numbers of bilayers are investigated. The thickness and growth rate of ZnO and TiO_2_ films are obtained using a spectroscopic ellipsometer, indicating the high accuracy of the ALD technique in controlling the growth of nanolaminates. The transmittance of multilayers in the visible wavelength increases gradually as the number of sample bilayers increases. The XRD spectra show that ZnO films grown on quartz are polycrystalline with preferred (002) orientation while TiO_2_ films are amorphous. The high-resolution TEM image for a representative sample shows clear lattice spacing along with the grain size of ZnO, confirming the structural properties of nanolaminated ZnO/TiO_2_ multilayers.

## Competing interests

The authors declare that they have no competing interests.

## Authors’ contributions

The experiments and characterization presented in this work were carried out by YZG, YG, and ZYY. The experiments were designed by YZG and HLL. YZG, YG, YZ, ZYY, QQS, SJD, HLL, and DWZ analyzed and discussed the results obtained from the experiments. The manuscript was prepared by YZG, and HLL helped with draft editing. All authors read and approved the final manuscript.
